# Distinct T cell polyfunctional profile in SARS-CoV-2 seronegative children associated with endemic human coronavirus cross-reactivity

**DOI:** 10.1016/j.isci.2023.108728

**Published:** 2023-12-14

**Authors:** Ntombi Benede, Marius B. Tincho, Avril Walters, Vennesa Subbiah, Amkele Ngomti, Richard Baguma, Claire Butters, Lina Hahnle, Mathilda Mennen, Sango Skelem, Marguerite Adriaanse, Heidi Facey-Thomas, Christiaan Scott, Jonathan Day, Timothy F. Spracklen, Strauss van Graan, Sashkia R. Balla, Thandeka Moyo-Gwete, Penny L. Moore, Rae MacGinty, Maresa Botha, Lesley Workman, Marina Johnson, David Goldblatt, Heather J. Zar, Ntobeko A.B. Ntusi, Liesl Zühlke, Kate Webb, Catherine Riou, Wendy A. Burgers, Roanne S. Keeton

**Affiliations:** 1 Institute of Infectious Disease and Molecular Medicine, University of Cape Town, Observatory, South Africa; 2 Division of Medical Virology, Department of Pathology, University of Cape Town, Observatory, South Africa; 3 Division of Paediatric Rheumatology, Department of Paediatrics and Child Health, Red Cross War Memorial Children’s Hospital, University of Cape Town, Observatory, South Africa; 4 Department of Medicine, University of Cape Town and Groote Schuur Hospital, Observatory, South Africa; 5 Department of Paediatrics and Child Health, University of Cape Town, Cape Town, South Africa; 6 Cape Heart Institute, Faculty of Health Sciences, University of Cape Town, Observatory, South Africa; 7 South African Medical Research Council, Francie Van Zijl Drive, Parow Cape Town, South Africa; 8 National Institute for Communicable Diseases of the National Health Laboratory Services, Johannesburg, South Africa; 9 SA MRC Antibody Immunity Research Unit, School of Pathology, University of the Witwatersrand, Johannesburg, South Africa; 10 Centre for the AIDS Programme of Research in South Africa, Durban, South Africa; 11 Medical Research Council (MRC) Unit on Child and Adolescent Health, University of Cape Town, Cape Town, South Africa; 12 Great Ormond Street Institute of Child Health Biomedical Research Centre, University College London, London, UK; 13 Wellcome Centre for Infectious Diseases Research in Africa, University of Cape Town, Observatory, South Africa; 14 Crick African Network, The Francis Crick Institute, London, UK

**Keywords:** Immunology, Immune response, Components of the immune system, Virology

## Abstract

SARS-CoV-2 infection in children typically results in asymptomatic or mild disease. There is a paucity of studies on SARS-CoV-2 antiviral immunity in African children. We investigated SARS-CoV-2-specific T cell responses in 71 unvaccinated asymptomatic South African children who were seropositive or seronegative for SARS-CoV-2. SARS-CoV-2-specific CD4^+^ T cell responses were detectable in 83% of seropositive and 60% of seronegative children. Although the magnitude of the CD4^+^ T cell response did not differ significantly between the two groups, their functional profiles were distinct, with SARS-CoV-2 seropositive children exhibiting a higher proportion of polyfunctional T cells compared to their seronegative counterparts. The frequency of SARS-CoV-2-specific CD4^+^ T cells in seronegative children was associated with the endemic human coronavirus (HCoV) HKU1 IgG response. Overall, the presence of SARS-CoV-2-responding T cells in seronegative children may result from cross-reactivity to endemic coronaviruses and could contribute to the relative protection from disease observed in SARS-CoV-2-infected children.

## Highlights

• SARS-CoV-2-specific T cell responses are detected in 60% of seronegative children
• Similar magnitude T cell response between seropositive and seronegative children
• Seronegative children exhibit a lower proportion of polyfunctional T cells
• CoV-2-specific T cell and endemic HKU1 IgG responses correlate in seronegative children

## Graphical abstract

**Figure:**
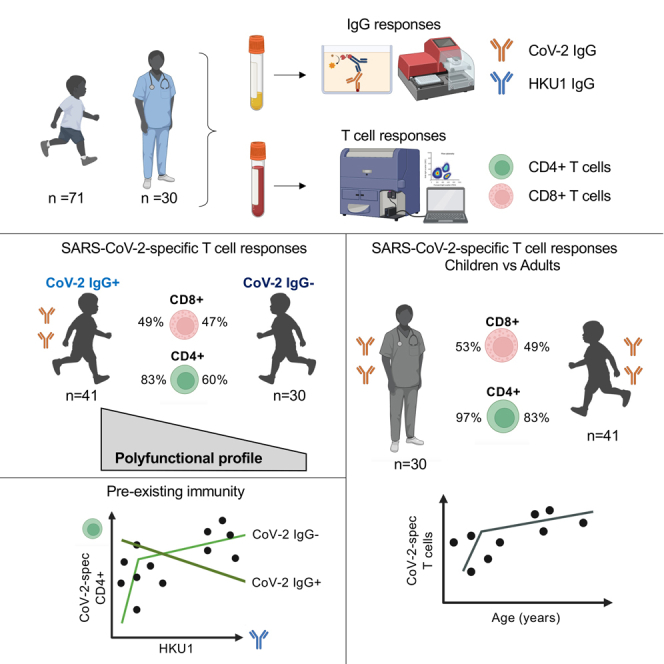

## Introduction

Severe Acute Respiratory Syndrome Coronavirus-2 (SARS-CoV-2) infection in children usually causes asymptomatic or mild illness, contrasting with the high rate of severe disease reported in older adults.^1^^,^^2^ As a result, global reports of coronavirus disease 2019 (COVID-19) cases among children and adolescents are underreported. The United Nations International Children’s Emergency Fund (UNICEF) estimates that 21% of all reported confirmed cases occur in individuals younger than 20 years.^3^

In the USA, it has been documented that COVID-19-associated hospitalization rates among children less than 18 years were lower compared to those in older individuals.^4^ Indeed, the COVID-19-Associated Hospitalization Surveillance Network (COVID-NET) reported that children younger than 18 years accounted for only 4.2% of COVID-19-associated hospitalization and 0.2% of COVID-19-associated in-hospital deaths.^5^ In South Africa, during the first 2 years of the pandemic, 12.5% of confirmed cases and 0.7% of COVID-19 associated in-hospital deaths were in individuals younger than 19 years.^6^

In contrast to the low COVID-19 severity in the majority of younger individuals, it is known that children are more susceptible than adults to other acute viral respiratory tract infections including respiratory syncytial virus (RSV), rhinovirus (RV), influenza virus and common circulating endemic human coronaviruses (HCoV).^7^ Several age-associated factors have been proposed to play a role in the reduction of severity to SARS-CoV-2 infection in children,^2^^,^^8^ including limited comorbidities,^9^^,^^10^^,^^11^ differences in the expression of SARS-CoV-2 viral entry factors,^12^^,^^13^^,^^14^ robust innate immune responses,^15^^,^^16^^,^^17^^,^^18^ humoral and cellular immunity^19^^,^^20^^,^^21^ and pre-existing immunity against common cold circulating endemic HCoVs.^22^^,^^23^^,^^24^^,^^25^^,^^26^ Endemic HCoVs account for 15 to 30% of respiratory infections reported annually in children.^27^^,^^28^ These HCoVs belong to the alpha-coronavirus subfamily (HCoV-229E and HCoV-NL63) and the beta-coronavirus subfamily (HCoV-OC43 and HCoV-HKU1), are generally considered to have seasonal infection peaks during the winter season and are responsible for high rates of infection among children.^29^^,^^30^ In contrast, the influenza surveillance data from India showed that HCoV-HKU1 peaked during spring to summer over the 2015–2016 period.^31^

It is well established that protective immune responses to SARS-CoV-2 encompass both an antibody and T cell component.^32^ Several studies have reported robust and durable antibody and T cell responses against SARS-CoV-2, which are maintained up to 6–12 months following infection.^21^^,^^33^^,^^34^^,^^35^^,^^36^ However, data on T cell responses in children compared to adults are conflicting, with studies reporting lower SARS-CoV-2-specific T cell responses in children,^23^^,^^37^^,^^38^ no differences^39^ or greater T cell responses in children.^22^ Many of these studies make use of ELISPOT assays which preclude analysis of T cell phenotypes or functional cytokine profiles.

In this study, we prospectively characterized specific T cell responses in SARS-CoV-2 seropositive and seronegative children during the COVID-19 pandemic and determined the functional profiles of SARS-CoV-2-specific T cells, in the context of their pre-existing immunity against endemic beta-HCoVs. This study provides insights into cross-reactive immunity to SARS-CoV-2 in children.

## Results

### Study cohort

**Figure 1:**
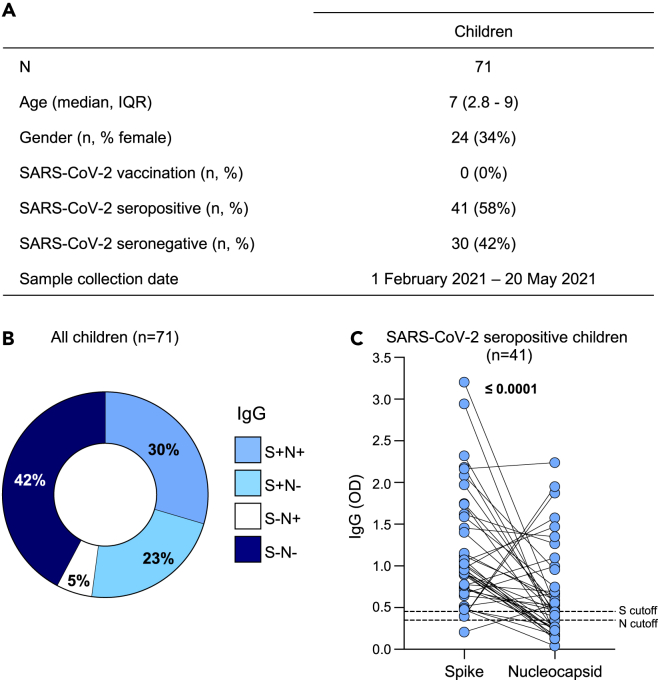
SARS-CoV-2-specific antibody responses in children (A) The demographic characteristics of 71 unvaccinated children included in this study. Age, gender, SARS-CoV-2 vaccination status, SARS-CoV-2 serology and collection date are shown. (B) Proportion of children exhibiting antibody responses to SARS-CoV-2 spike (S) and nucleocapsid (N) proteins. (C) The magnitude of SARS-CoV-2 S and N IgG antibodies (OD_490nm_) measured by ELISA in seropositive children (n = 41). The dotted lines indicate the cut-off for positivity which was calculated as the mean optical density of COVID-19 prepandemic control samples. Statistical analysis was performed using the Wilcoxon signed rank test; p values < 0.05 were considered statistically significant and are bolded. See also Figure S1.

To investigate SARS-CoV-2-specific immune responses, we measured immunoglobulin (Ig) G and T cell responses in 71 healthy children recruited in Cape Town, Western Cape, South Africa. The participants are described in Figure 1A. The median age of the children was 7 years (interquartile range (IQR) 2.8–9 years) and 34% (24/71) were female. The children included in this study had not received any SARS-CoV-2 vaccine prior to recruitment and no PCR-confirmed infection data were available, although 58% showed SARS-CoV-2 seropositivity (defined as being positive for either anti-spike or anti-nucleocapsid IgG). Samples were collected between 1 February 2021 and 20 May 2021, after two infection waves in South Africa, that were dominated by the ancestral D614G strain followed by the Beta variant of concern.^40^

### SARS-CoV-2-specific antibody responses in children

To characterize the children serologically, we measured IgG responses against SARS-CoV-2 spike and nucleocapsid proteins using an indirect Enzyme-linked immunosorbent assay (ELISA) (Figure 1B). A large proportion (58%, 41/71) of unvaccinated children had detectable SARS-CoV-2-specific IgG against spike and/or nucleocapsid proteins and were classified as seropositive. Of the seropositive children, 51% (21/41) had spike- and nucleocapsid-specific IgG, 39% (16/41) had only spike-specific IgG and 10% (4/41) had only nucleocapsid-specific IgG only (Figure 1B). The remaining 42% (30/71) had undetectable SARS-CoV-2-spike and nucleocapsid-specific IgG and were classified as seronegative (Figure 1B). IgG responses to spike and nucleocapsid are shown for the 41 seropositive children in Figure 1C. Moreover, we compared SARS-CoV-2-spike- and nucleocapsid-specific IgG levels among SARS-CoV-2 seropositive (n = 41) children stratified by age. Children under the age of 5 years (n = 13) had significantly higher SARS-CoV-2-spike-specific IgG responses (p = 0.0007) compared to children older than 5 years old (n = 28) (Figure S1A). However, no significant differences (p = 0.972) were observed for SARS-CoV-2-nucleocapsid-specific IgG responses (Figure S1B).

### SARS-CoV-2-specific T cell responses in children

**Figure 2:**
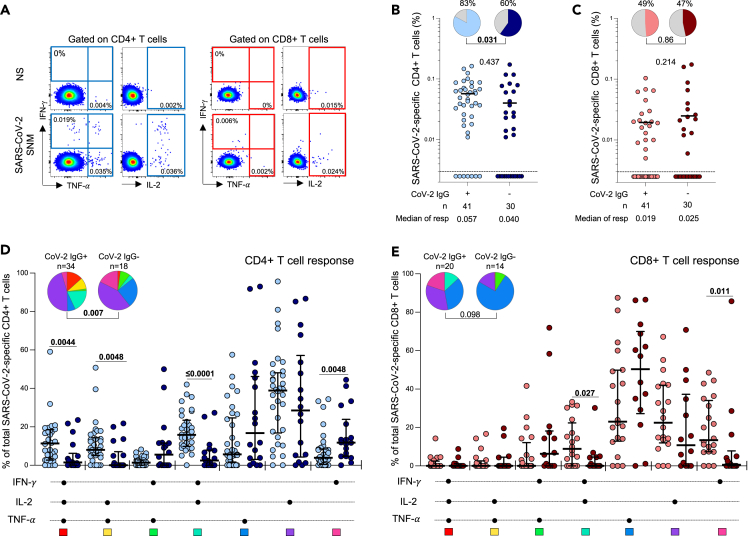
SARS-CoV-2-specific T cell responses in children (A) Representative flow cytometry plots of SARS-CoV-2-specific interferon-γ (IFN-γ), tumor necrosis factor-α (TNF-α) and interleukin-2 (IL-2) cytokine production from CD4^+^ (left) and CD8^+^ (right) T cells in response to SARS-CoV-2 peptide stimulation. NS: no stimulation, SNM: combined peptide pool of SARS-CoV-2-spike, nucleocapsid and membrane proteins. (B) Frequency of SARS-CoV-2-specific CD4^+^ T cells producing any of the measured cytokines (IFN-γ, IL-2 or TNF-α). Children were grouped according to SARS-CoV-2 serostatus (light blue: n = 41 SARS-CoV-2 seropositive; dark blue: n = 30 SARS-CoV-2 seronegative). Bars represent the median of the responders, and median values are indicated. The pie charts represent the proportion of responders with detectable T cell response to SARS-CoV-2 SNM peptides. (C) Frequency of SARS-CoV-2-specific CD8^+^ T cells producing any of the measured cytokines (IFN-γ, IL-2 or TNF-α). Children were grouped according to SARS-CoV-2 serostatus (light red: n = 41 SARS-CoV-2 seropositive children; dark red: n = 30 SARS-CoV-2 seronegative children). Statistical comparisons in (B) and (C) were performed using the Mann-Whitney test between seropositive and seronegative children and the Chi-square test to compare the percentage of responders; p values < 0.05 were considered statistically significant and are bolded. (D) Polyfunctional profile of SARS-CoV-2-specific CD4^+^ and (E) CD8^+^ T cells in seropositive and seronegative unvaccinated children. The x axis illustrates each combination which is indicated with a black circle for the presence of IFN-γ, IL-2 and TNF-α. The medians and interquartile range are shown. Each response pattern (any possible combination of IFN-γ, IL-2 and TNF-α production) is color coded and summarized in the pie charts, with each pie slice representing the median contribution of each combination to the total SARS-CoV-2 responses. The permutation test was used to compare the statistical differences between the pie charts and the Mann Whitney Sum Test to compare response patterns between seropositive and seronegative children; p values < 0.05 were considered statistically significant and are bolded.

The magnitude of SARS-CoV-2-specific T cell responses in children was quantified using a whole blood assay and intracellular cytokine staining followed by flow cytometry. SARS-CoV-2-specific CD4^+^ and CD8^+^ T cell responses were measured as the total cytokine production of interferon-γ (IFN-γ), tumor necrosis factor-α (TNF-α) or interleukin-2 (IL-2) in response to a combined peptide pool covering SARS-CoV-2 spike (S), nucleocapsid (N) and membrane (M) proteins (Figure 2A).

We assessed the proportion of responders and magnitude of SARS-CoV-2-specific T cell responses according to the serostatus of the children (Figures 2B and 2C). As expected, 83% (34/41) of seropositive children had detectable SARS-CoV-2-specific CD4^+^ T cells. Interestingly, 60% (18/30) of seronegative children also had detectable SARS-CoV-2-specific CD4^+^ T cells, although this was a significantly lower proportion than observed in the seropositive group (p = 0.031). The magnitude of the SARS-CoV-2-specific CD4^+^ T cell response in seronegative responders was comparable to that observed in seropositive children (median of responders: 0.04% and 0.057% respectively; p = 0.437, Figure 2B). Conversely, we did not observe significant differences in the proportion of SARS-CoV-2 CD8^+^ T cell responders (∼50%) or the frequency of SARS-CoV-2-specific CD8^+^ T cells between seropositive and seronegative children (median of responders: 0.019% and 0.025% respectively; p = 0.214, Figure 2C).

Next, we evaluated the polyfunctional profile of SARS-CoV-2-responding CD4^+^ and CD8^+^ T cells based on the co-production of IFN-γ, IL-2 or TNF-α (Figures 2D and 2E). The overall functional profile of SARS-CoV-2-specific CD4^+^ T cells in seropositive children was distinct from that of seronegative children (p = 0.007, Figure 2D). SARS-CoV-2-specific CD4^+^ T cells in seropositive children were more polyfunctional, exhibiting a higher proportion of triple functional IFN-γ+IL-2+TNF-α+-producing CD4^+^ T cells (p = 0.0044), and dual expressing cells (IL-2+TNF-α+, p = 0.0048; IFN-γ+IL-2+, p ≤ 0.0001) compared to the seronegative children. In contrast, seronegative children were characterized by an increased proportion of IFN-γ monofunctional CD4^+^ T cells (p = 0.0048). Considering the CD8 compartment, we detected no significant differences in the overall functional profile of SARS-CoV-2-responding CD8^+^ T cells between the two groups (p = 0.098), although seropositive children did have a larger proportion of CD8^+^ T cells producing both IFN-γ and IL-2 (p = 0.027) and single IFN-γ (p = 0.011) (Figure 2E).

Taken together, these data show that a significant proportion of seronegative children have detectable SARS-CoV-2-specific CD4^+^ and CD8^+^ T cells. Notably, these CD4^+^ T cells exhibit a distinct polyfunctional cytokine profile compared to those found in SARS-CoV-2-exposed children.

### Pre-existing immunity to endemic common circulating human coronaviruses

**Figure 3:**
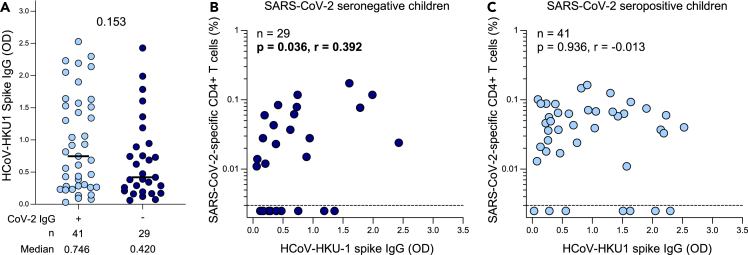
SARS-CoV-2 cross-reactivity to endemic beta-HCoV in children (A) The magnitude of HCoV-HKU-1 spike IgG levels were measured by ELISA in SARS-CoV-2 seropositive (light blue; n = 41) and seronegative (dark blue; n = 29) children. Plasma sample was insufficient for one seronegative child therefore OD for HCoV-HKU1 was not measured for this participant. The bars represent the median values. A statistical comparison was performed using the Mann-Whitney test between seropositive and seronegative children; a p value < 0.05 was considered statistically significant. (B) Correlation between the frequency of SARS-CoV-2-specific CD4^+^ T cells and HCoV-HKU-1-spike IgG levels in SARS-CoV-2 seronegative children (n = 29). One participant had insufficient sample available to be included in this assay. (C) Correlation between the frequency of SARS-CoV-2-specific CD4^+^ T cells and HCoV-HKU-1-spike IgG levels in SARS-CoV-2 seropositive children (n = 41). Statistical comparisons for (B) and (C) were performed using a two-tailed non-parametric Spearman rank tests; p values < 0.05 were considered statistically significant and are bolded and correlation coefficients values are shown. See also Figure S2.

The presence of SARS-CoV-2-responding CD4^+^ T cells primarily exhibiting a monofunctional profile observed in seronegative children led us to hypothesize that the T cell response might be due to cross-reactivity resulting from prior infection with common circulating endemic HCoVs. Therefore, we measured endemic beta HCoV-HKU1 and HCoV-OC43 spike IgG in SARS-CoV-2 seropositive and seronegative children. The magnitude of both HCoV-HKU1 (Figure 3A) and HCoV-OC43 (Figure S2A) spike IgG was comparable in the seropositive and seronegative groups (median OD: 0.746 vs. 0.420; p = 0.153 for HCoV-HKU1 and 1.769 vs. 1.281; p = 0.07 for HCoV-OC43, respectively).

We found a moderate correlation between the frequency of SARS-CoV-2-specific CD4^+^ T cells and HCoV-HKU1-spike IgG in seronegative children (p = 0.036, r = 0.392, Figure 3B), while no correlation was observed in seropositive children (p = 0.936, r = −0.013, Figure 3C). For HCoV-OC43, no correlation was detected in either the seronegative (p = 0.324, r = −0.187, Figure S2B) or the seropositive group (p = 0.171, r = −0.218, Figure S2C). Overall, these results may suggest the SARS-CoV-2-reactive CD4^+^ T cell responses in a proportion seronegative children could be due in part to cross-reactive T cell immunity resulting from prior infection with common cold HCoV-HKU1.

### SARS-CoV-2-specific T cell responses in SARS-CoV-2 seropositive children compared to COVID-19 convalescent adults

**Figure 4:**
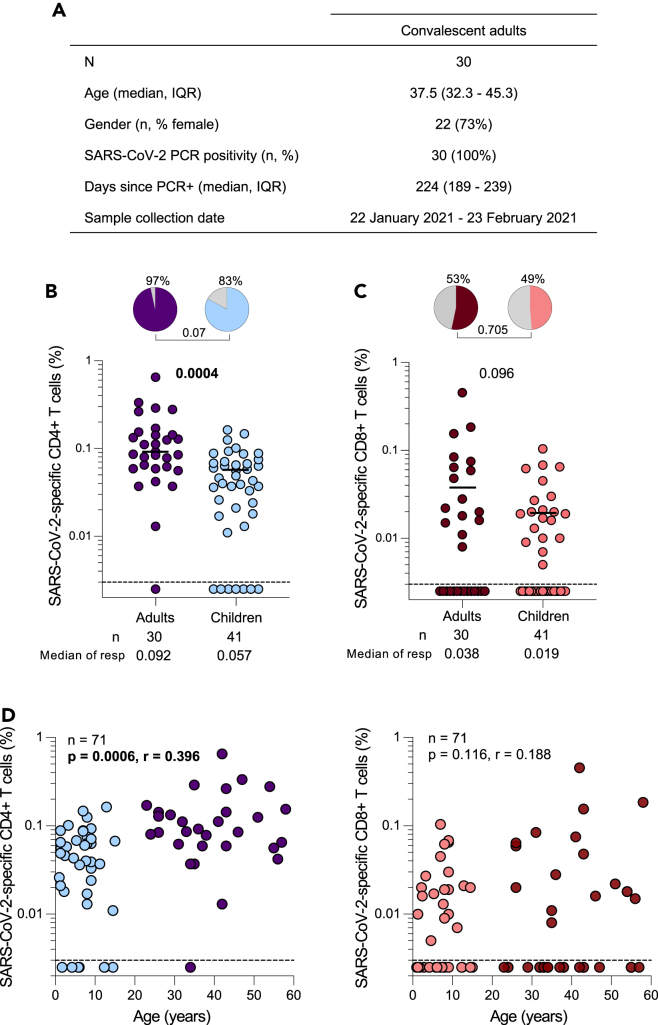
SARS-CoV-2-specific T cell responses in children compared to convalescent adults (A) The demographic characteristics of 30 unvaccinated convalescent adults included in this study. Age, sex, SARS-CoV-2 PCR-positivity, days since PCR test and collection date are shown. (B) Frequency of SARS-CoV-2-specific CD4^+^ T cells producing any of the measured cytokines (IFN-γ, TNF-α, or IL-2) in SARS-CoV-2 convalescent HCW (purple; n = 30) and seropositive children (light blue; n = 41). The pie charts represent the proportion of responders with a detectable T cell response to SARS-CoV-2 SNM combined peptide pools. Bars represent median of the responders. (C) Frequency of SARS-CoV-2-specific CD8^+^ T cells producing any of the measured cytokines (IFN-γ, TNF-α, or IL-2) in SARS-CoV-2 convalescent HCW (dark red; n = 30) and seropositive children (light red; n = 41). The bars represent the median of the responders and median values are indicated. Statistical comparisons were performed using the Mann-Whitney test between seropositive children and adults and the Chi-square test was used to compare the percentage of responders for (B) and (C); p values < 0.05 were considered statistically significant and are bolded. (D) Correlations between SARS-CoV-2-specific CD4^+^ or CD8^+^ T cells and age in convalescent HCW (purple and dark red; n = 30) and seropositive (light blue and light red; n = 41) children. Statistical comparisons were performed using a two-tailed non-parametric Spearman rank tests; p values < 0.05 were considered statistically significant and are bolded and correlation coefficients are shown.

Blood and plasma samples were collected from 30 COVID-19 convalescent healthcare workers (HCWs) participating in a longitudinal study at Groote Schuur Hospital (Cape Town, Western Cape, South Africa) (Figure 4A). All convalescent adults had a prior mild/asymptomatic PCR-confirmed SARS-CoV-2 infection (median 224 days prior to sampling) and had not received a COVID-19 vaccine at the time of sampling. Samples were collected between 22 January 2021 and 23 February 2021.

A trend toward a higher proportion of SARS-CoV-2 CD4 responders was observed in convalescent adults (97%) compared to seropositive children (83%) (p = 0.07, Figure 4B). Moreover, in SARS-CoV-2 responders, convalescent adults did have a significantly higher frequency of SARS-CoV-2-specific CD4^+^ T cells compared to seropositive children (median of responders: 0.092% vs. 0.057%; p = 0.0004, respectively). Unlike CD4^+^ T cell responses, there were no significant differences in either the proportion of CD8 responders (53% vs. 49%, p = 0.705) or the frequency of SARS-CoV-2-specific CD8^+^ T cells (median of responders: 0.038% vs. 0.019%; p = 0.096) between convalescent HCWs and seropositive children (Figure 4C). Furthermore, we observed a moderate correlation between age and the frequency of SARS-CoV-2-specific CD4^+^ T cells (p = 0.0006, r = 0.396, Figure 4D). No correlation was observed with SARS-CoV-2-specific CD8^+^ T cells and age (p = 0.116, r = 0.188, Figure 4D). Overall, these results show that convalescent adults mount higher SARS-CoV-2-specific CD4^+^ T cell responses compared to children. This disparity may, in part be attributed to the incomplete maturation of the adaptive immune system in children.^41^^,^^42^

## Discussion

T cells have been associated with protection from severe COVID-19.^43^ It is therefore important to understand the nature of T cell responses targeting SARS-CoV-2 in children, who have been largely spared from severe COVID-19. In this study, comparing SARS-CoV-2-specific T cell responses in SARS-CoV-2 seropositive and seronegative children, a sizable proportion of seronegative children had SARS-CoV-2-reactive CD4^+^ and CD8^+^ T cells, but their CD4^+^ T cells exhibited a distinct functional cytokine profile compared to seropositive children. Importantly, in seronegative children, the frequency of SARS-CoV-2-reactive CD4^+^ T cells positively associated with HCoV-HKU1 spike-specific IgG antibodies. Additionally, we showed that convalescent adults had a higher magnitude of CD4^+^ T cell responses against SARS-CoV-2 compared to seropositive children, which associated positively with age.

Our data are in accordance with several studies showing that children develop robust humoral and cellular immunity to SARS-CoV-2 infection^20^^,^^22^^,^^39^^,^^44^^,^^45^ and BNT162b2 vaccine.^46^ In addition, SARS-CoV-2-specific T cell responses have been detected in 40–60% of SARS-CoV-2 unexposed individuals including children and adults^22^^,^^23^^,^^47^^,^^48^^,^^49^^,^^50^^,^^51^^,^^52^ suggesting possible cross reactivity to HCoVs. One study demonstrated that the proportion of T cell responders was higher in children than adults.^22^ In contrast, a study by Tsang et al. found that SARS-CoV-2 uninfected children failed to mount detectable T cell responses.^44^ A further study showed that only a small proportion of seronegative children mounted a SARS-CoV-2-specific CD4^+^ T cell response (13%) compared to 60% in seropositive siblings, despite similar exposure in shared households.^45^ These two studies differ from our findings, where we showed that 60% of seronegative children had detectable SARS-CoV-2-specific T cell responses, consistent with Dowell et al.^22^ The discrepancies between the studies could be due to a difference in the seasonal prevalence of circulating HCoVs in each study setting,^29^^,^^30^^,^^53^ different T cell assays used to analyze T cell responses, and/or the cohort demographics.

Although the source of SARS-CoV-2 reactive T cells in seronegative children remains unclear, mounting evidence argues for cross-reactivity to endemic HCoVs. HCoVs (including beta HCoVs-HKU1 and OC43, and alpha HCoVs-NL63 and 229E) have partial sequence homology with SARS-CoV-2.^52^^,^^54^ While cross reactive humoral responses have been shown not to underly this protection,^55^ studies have reported pre-existing T cell responses to endemic HCoVs with cross-reactivity to SARS-CoV-2 in unexposed adults^50^^,^^56^^,^^57^^,^^58^^,^^59^^,^^60^^,^^61^ and children.^22^^,^^24^^,^^62^^,^^63^ These cellular responses were targeted mainly toward the S2 subunit which is highly conserved among coronaviruses.^22^^,^^25^ In our study, we showed a positive correlation between the frequency of SARS-CoV-2 CD4^+^ T cells and the magnitude of HKU1 spike-specific IgG in SARS-CoV-2 seronegative children. Our findings are supported by similar findings showing that 58% (7/12) seronegative children had binding antibodies against alpha and/or beta HCoV spike proteins, which they concluded were likely due to recent HCoV infection.^22^

Pre-existing immunity from endemic HCoV infection or exposure has been shown to be associated with a protective effect against COVID-19 disease in adults.^56^^,^^59^^,^^60^^,^^61^^,^^64^ Sagar et al. showed that individuals with a documented recent or ongoing HCoV infection and SARS-CoV-2 infection had fewer COVID-19-associated complications when hospitalized, compared to individuals without HCoV infection.^64^ Previously, the clinical relevance of pre-existing immunity and cross-reactive cellular responses from endemic HCoVs in providing protection from SARS-CoV-2 infection in children was unclear. Recently however, Dowell et al. demonstrated that SARS-CoV-2-reactive T cell responses in SARS-CoV-2 seronegative children were associated with relative protection against Omicron infection, suggesting a protective role in SARS-CoV-2 unexposed children.^65^

Polyfunctional T cells have been reported to be associated with protection against several viral diseases.^66^^,^^67^^,^^68^^,^^69^ These include protection against cytomegalovirus (CMV) infection after lung transplantation,^67^ improved viral control of hepatitis C virus (HCV)^68^ and detection during human immunodeficiency virus (HIV) infection in long term non-progressors.^69^ We found that seropositive children had a more polyfunctional profile of SARS-CoV-2-specific CD4^+^ T cells while CD4 responses in seronegative children exhibited a predominantly monofunctional profile. The SARS-CoV-2-specific polyfunctional Th1 CD4 T cell response (characterized by the co-expression of IFN-γ, TNF-α and/or IL-2), as seen in seropositive children, may be necessary for effective viral control and has been documented in COVID-19 convalescent adults.^70^^,^^71^

The distinct CD4 and CD8 polyfunctional profiles reported in our findings could be a result of differing degrees of memory T cell differentiation and/or lower SARS-CoV-2 peptide binding affinity with HCoV-specific T cells resulting in low T cell activation. The affinity between MHC molecules presenting peptides (pMHC) and the T cell receptor (TCR) plays a role in antigen recognition. It has been reported high TCR-pMHC complex affinity is associated with increased overall T cell functional efficacy *in vitro* and *in vivo* and therefore an increased polyfunctional profile compared to low affinity TCRs.^72^^,^^73^^,^^74^ TCR cross-recognition of pMHCs that are not structurally identical results in lower binding affinity which decreases T cell polyfunctionality.^73^^,^^75^ It is therefore plausible that the distinct polyfunctional profile of SARS-CoV-2 CD4^+^ T cell responses in seronegative children observed in our study may be mediated by the cross-recognition of pre-existing T cell immunity to HCoV-HKU1, where partial sequence homology between HCoV-HKU1 and SARS-CoV-2 results in low peptide binding affinity and a different polyfunctional T cell profile.^52^^,^^58^^,^^72^^,^^75^^,^^76^ It is noteworthy to report that we did not find any association with the other beta HCoV-OC43 spike-specific IgG and SARS-CoV-2-specific T cells in seronegative children, which could be related to differences in seasonal prevalence of types of HCoV infections. Indeed, multiple studies have shown differences in the prevalence of HCoV infections which change yearly and depend on regional environment.^31^^,^^77^^,^^78^^,^^79^^,^^80^ In particular, a study by Friedman et al. showed a high prevalence of HCoV-OC43 was the most prevalent HCoV in winter but HCoV-HKU1 was the most prevalent in spring to summer during the 2015–2016 time period.^31^

Numerous studies have now compared the magnitude of the SARS-CoV-2 specific T cell response in children to those detectable in adults.^22^^,^^23^^,^^37^^,^^38^^,^^39^^,^^44^ Initially it was thought that children may have a higher magnitude of T cell responses given their relative resistance to severe disease and the link between T cell responses and protection from severe disease.^8^^,^^81^ However, children were shown to have lower T cell responses than adults in most studies, including the current study.^23^^,^^37^^,^^38^ Several hypotheses could possibly explain the differences in the magnitude of SARS-CoV-2-specific CD4^+^ T cell responses between children and adults in our study. These include 1) differences in disease severity between the groups, 2) the immaturity of children’s immune system and 3) the timing of infection in relation to sampling. Adults have a mature immune system with more differentiated memory T cell subsets endowed with increased cytokine capacity, whereas children have an immature immune system, with many more naive T cells which have reduced cytokine producing capacity, are enriched for monofunctional responses, and have increased antigen dependence.^82^ However, as the exact time of SARS-CoV-2 infection in the children group could not be defined, we cannot fully exclude that the higher magnitude of SARS-CoV-2-specific CD4^+^ T cells observed in the adult group could be related to a more recent infection compared to the children group. Despite lower circulating SARS-CoV-2-specific T cells, children and adults may also have different responses in the respiratory tract. There is evidence in adults demonstrating the presence of SARS-CoV-2-specific T cell responses in the nasal mucosa after infection and vaccination.^83^^,^^84^ However, comparative studies have yet to be performed in children. Additionally, several studies have proposed that one potential contributing factor to age-related COVID-19 clinical outcome is the robust innate immune responses observed in children, which may contribute to early control of viral replication.^15^^,^^16^^,^^17^^,^^85^ Recent studies have shown that, compared to adults, SARS-CoV-2 seronegative children exhibited an increased number of innate immune cells with pre-activated signatures leading to the early production of interferon-mediated antiviral effects in the upper respiratory tract.^18^^,^^19^

In conclusion, our study shows that a robust SARS-CoV-2-specific T cell response is observed in children, including those with no evidence of prior SARS-CoV-2 infection. We demonstrate that the magnitude of SARS-CoV-2-reactive CD4^+^ T cells in seronegative children correlates with HCoV-HKU1 exposure. This, together with a distinct functional profile of SARS-CoV-2-specific responding CD4^+^ T cells observed between seropositive and seronegative children may provide further evidence for pre-existing T cell responses cross-reactive to SARS-CoV-2.

### Limitations of the study

This study relied on serology to determine SARS-CoV-2 exposure in the pediatric cohort. With no PCR confirmation of SARS-CoV-2 infection, the exact time of infection of the children could not be determined. This caveat in our study may have an impact in the differences of SARS-CoV-2-specific T cell responses seen in adults compared to seropositive children. Additionally, our study investigated T cell responses against SARS-CoV-2 spike, nucleocapsid and membrane proteins, and not the non-structural viral proteins that can also serve as targets for the T cell response, thus not capturing the full extent of T cell reactivity in infected participants. While our study focused solely on Th1-associated cytokines, it remains to define whether other Th CD4 subtypes such Th2 or Th17 could as also play a role in COVID-19 protection. Furthermore, we did not address durability of the T cell responses in children due to the cross-sectional design of the study. Moreover, it would be of interest to assess the HCoV-HKU1-specific memory T cell responses and define the relationship with SARS-CoV-2 specific immune responses and their potential protective effect against SARS-CoV-2. Further experiments assessing HCoV-IgG responses as well as the neutralizing antibody activity against HCoVs, SARS-CoV-2 and its variants of concern are warranted to gain a better understanding of the relative protection from disease observed in SARS-CoV-2-infected children compared to adults.

## STAR★Methods

### Key resources table

**Table:** 

REAGENT or RESOURCE	SOURCE	IDENTIFIER
**Antibodies**		

Purified NA/LE mouse anti-human CD28 (clone 28.2)	BD Pharmingen	Cat# 555725; RRID:AB_2130052
Purified NA/LE mouse anti-human CD49d (clone L25)	BD Pharmingen	Cat# 555501; RRID:AB_396068
CD4 ECD (clone SFCI12T4D11)	Beckman Coulter	Cat# 6604727; RRID:AB_394488
CD8 BV510 (clone RPA-8)	Biolegend	Cat# 301048; RRID:AB_2561942
CD3 BV650 (clone OKT3)	Biolegend	Cat# 317324; RRID:AB_2563352
IFN-g AlexaFluor® 700 (clone B27)	BD Biosciences	Cat# 557995; RRID:AB_396977
TNF-a BV786 (clone Mab11)	Biolegend	Cat# 502948; RRID:AB_2565858
IL-2 APC (clone MQ1-17H12)	Biolegend	Cat# 500310; RRID:AB_315097
Anti-human IgG (Fab-specific)-horseradish peroxidase	Sigma Aldrich	Cat# A0170; RRID:AB_257868

**Biological samples**		

Unvaccinated children blood samples	Red Cross War Memorial Children’s Hospital	https://www.childrenshospitaltrust.org.za/the-hospital/
Unvaccinated children blood samples	Mbekweni and TC Newman clinics	https://www.westerncape.gov.za/facility/mbekweni-community-day-centre-cdc https://www.westerncape.gov.za/facility/tc-newman-community-day-centre-cdc
Convalescent health care worker blood samples	Groote Schuur Hospital	https://www.gsh.co.za

**Chemicals, peptides, and recombinant proteins**		

Brefeldin A	Sigma Aldrich	Cat# B7651-5MG
PepTivator® SARS-CoV-2 Prot_S	Miltenyi Biotech	Cat # 130-126-701
Peptide Array, SARS-Related Coronavirus 2 Nucleocapsid (N) Protein	BEI resources	Cat# 52404
Peptide Array, SARS-Related Coronavirus 2 Membrane (M) Protein	BEI resources	Cat# 52403
PtX™ SARS-CoV-2 Spike Protein (S1, Rabbit FC)	Cape Bio Pharms (Pty) Ltd	Cat# CB_0002.2
Recombinant SARS-CoV-2 Spike RBD His-tag (HEK293 Expressed)	Bio-Techne	Cat#10500-CV
BTA SARS-CoV-2 Nucleocapsid (1mg)	BioTech Africa	Cat# BA25-C
Recombinant HCoV-HKU1 spike (HEK293F Expressed)	In-house	N/A
Recombinant HCoV-OC43 spike (HEK293F Expressed)	In-house	N/A
O-phenylenediamine dihydrochloride (OPD), Sigmafast TM	Sigma Aldrich	P9187-50SET
3′,5,5′-Tetramethylbenzidine (TMB) substrate	Thermofisher Scientific	Cat# 34029
Casein, Hammarsten Bovine	Sigma Aldrich	E0789-500
Phosphate buffered saline with 0.05% tween, pH 7.4	Sigma Aldrich	P3563-10PAK

**Critical commercial assays**		

BD FACS™ Lysing Solution	BD Biosciences	Cat # 349202
BD Perm/Wash Buffer	BD Biosciences	Cat# 554723
CellFIX buffer	BD Biosciences	Cat# 340181

**Experimental models: Cell lines**		

Human Embryonic Kidney (HEK) 293F	Dr Nicole Doria-Rose, VRC, USA	N/A

**Recombinant DNA**		

HCoV-HKU1 and -OC43 spike plasmids	Dr Gaurav Kwatra and Prof Shabir A. Madhi, Wits VIDA, SA Prof Barney Graham, VRC, USA	N/A

**Software and algorithms**		

FACSDiva 9	BD Biosciences	https://www.bdbiosciences.com
FlowJo 10	FlowJo, LLC	https://www.flowjo.com
Graphpad Prism 9	Graphpad	https://graphpad.com
BioRender	BioRender	https://biorender.com

### Resource availability

#### Lead contact

Further information and requests for resources and reagents should be directed to and will be fulfilled by the lead contact, Roanne S. Keeton (roanne.keeton@uct.ac.za).

#### Materials availability

Requests for materials should be directed to and will be fulfilled by the lead contact, Roanne S. Keeton (roanne.keeton@uct.ac.za).

#### Data and code availability

• All data generated in this study is included in the accompanying tables, figures, and supplemental materials.
• No code was generated in this study.
• Any additional information required to reanalyse the data reported in this paper is available from the lead contact Roanne S. Keeton (roanne.keeton@uct.ac.za) upon request.

### Experimental model and subject details

#### Study participants

Pediatric participants (n = 71) were recruited from two cohorts in the Western Cape Province, South Africa. The first cohort enrolled 50 children from the Red Cross War Memorial Children’s Hospital (Cape Town, Western Cape, South Africa). In this cohort 50/71 children were hospitalized for non-COVID-19-related elective procedures and were confirmed to be clinically well, with no chronic or intercurrent infectious or inflammatory disease. This study is approved by University of Cape Town Human Research Ethics Committee as a Pediatric Immunology Biorepository (UCT HREC 112/2012) and MIS-C substudy (UCT HREC 599/2020). A further 21 children were enrolled from the Drakenstein Child Health Study (DCHS) (Cape Winelands Western Cape, South Africa), a birth cohort study.^86^ The participants from this study were recruited between 1 February 2021 and 20 May 2021, after the first and the second infection waves with Ancestral strain and Beta variant in South Africa.^87^ The study was approved by the University of Cape Town Human Research Ethics Committee and renewed annually (HREC 401/2009). Parents or legal guardians provided written informed consent for all pediatrics participants.

COVID-19 convalescent unvaccinated adults from a longitudinal study of healthcare workers (HCW) enrolled from Groote Schuur Hospital (Cape Town, Western Cape, South Africa) were included in the study (n = 30). HCW in this cohort were recruited between July 2020 and January 2021 and were selected for inclusion based on a prior PCR-confirmed SARS-CoV-2 infection at least 3 months earlier. All participants were asymptomatic or had mild symptoms and did not require hospitalization for COVID-19 and were symptom-free at the time of sampling. Written informed consent was obtained from all participants and the study was approved by the University of Cape Town Human Research Ethics Committee (HREC 190/2020 and 209/2020).

### Methods details

#### SARS-CoV-2 and HCoV antigens

For serology assays, recombinant SARS-CoV-2 spike S1 (Cape Bio Pharms), and SARS-CoV-2 nucleocapsid (BioTech Africa) proteins were used for this study.

HKU1 and OC43 spike proteins were expressed in Human Embryonic Kidney (HEK) 293F suspension cells by transfecting the cells with the spike plasmid. After 6 days, proteins were purified using a nickel resin followed by size-exclusion chromatography. Relevant fractions were collected and frozen at −80°C until use.

SARS-CoV-2 peptides used for T cell assays included a commercially available peptide pool (15mers peptides with 11 amino acid overlap) covering the immunodominant regions of SARS-CoV-2 spike protein (PepTivator SARS-CoV-2 Prot_S, Miltenyi Biotech) based on the Wuhan-1 strain. The Spike peptide pool was prepared by resuspending in distilled water and used at a final concentration of 1 μg/mL. SARS-CoV-2 (Wuhan-1) nucleocapsid and membrane peptides (17mers with 10 amino acid overlap spanning the full proteins) were obtained from BEI Resources and were prepared by resuspending in dimethyl sulfoxide (DMSO, Sigma) and used at a concentration of 1 μg/mL.

#### Enzyme-linked immunosorbent assay (ELISA)

SARS-CoV-2-specific enzyme-linked immunosorbent assay (ELISA) was performed to characterize the serostatus of participants. Two ug/mL of spike or nucleocapsid protein was used to coat 96-well high-binding plates and incubated overnight at 4°C. The plates were incubated in a blocking buffer consisting of 1% casein, 0.05% Tween 20, 1× Phosphate-Buffered Saline (PBS) for SARS-CoV-2 or 1× PBS, 5% skimmed milk powder, 0.05% Tween 20 for HCoVs for 1 h. Plasma samples were diluted to 1:50 for SARS-CoV-2 or 1:100 for HCoVs in the respective blocking buffer and added to the plates for 2 h. For the SARS-CoV-2 ELISA, secondary antibody was diluted to 1:5000 in dilution buffer and added to the plates for 1 h followed by SigmaFast O-phenylenediamine dihydrochloride (OPD) substrate for 12 min. For the HCoV ELISAs, secondary antibody was diluted to 1:3000 in blocking buffer and added to the plates followed by TMB substrate (Thermofisher Scientific). Upon stopping the reaction with 1–3 M sulfuric acid, absorbance was measured at a wavelength of 490 nm for SARS-CoV-2 or 450 nm for HCoVs. A cut-off for positivity was set at two standard deviations (SD) above the mean optical density (OD) of prepandemic samples (n = 40) for SARS-CoV-2 ELISAs.

#### Whole blood-based T cell assay

Blood was collected in sodium heparin tubes and processed within 4–6 h of collection. The whole blood assay sample processing used for this study was adapted from a whole blood intracellular cytokine detection assay designed to detect SARS-CoV-2 specific T cells in adults.^88^^,^^89^ For this study, 500 μL of blood was stimulated for 24 h at 37°C with a combined pool of SARS-CoV-2 peptides including S, N and M, all at 1 μg/mL in the presence of costimulatory antibodies against CD28 (clone 28.2) and CD49d (clone L25) (1 μg/mL each; BD Biosciences) and Brefeldin A (10 μg/mL, Sigma-Aldrich). Unstimulated blood was incubated with costimulatory antibodies, Brefeldin A and an equimolar amount of DMSO as a background control. After 24 h, blood was treated with EDTA (2 mM) for 15 min followed by red blood cell lysis and white cell fixation using FACS lysing solution (BD Biosciences) for 10 min. Cells were then cryopreserved in freezing media (90% fetal bovine serum (FBS) and 10% DMSO) and stored at −80°C until batched analysis.

#### Cell staining and flow cytometry

Cell staining was performed on cryopreserved fixed cells that were thawed and washed with 1% FACS washing buffer (1% FBS in PBS). A viability dye was not included during the staining process, as it has been reported that the omission of a viability dye during the staining process does not impact the detection and quantification of cytokines when using the whole blood-based T cell assay.^88^ Cells were stained with the following surface antibody markers: CD4 ECD (SFCI12T4D11, Beckman Coulter), CD8 BV510 (RPA-8, Biolegend) and incubated at room temperature for 20 min. Cells were permeabilized and stained with intracellular antibody markers CD3 BV650 (OKT3), IFN-γ AlexaFluor 700 (B27), TNF-α BV786 (Mab11) and IL-2 APC (MQ1-17H12) (all from Biolegend). Finally, cells were washed and fixed with Cellfix (BD Biosciences). Samples were acquired on a multiparameter BD Fortessa flow cytometer using Diva software version 9 and analyzed using FlowJo v10. Results are expressed as the frequency of CD4^+^ or CD8^+^ T cells expressing IFN-γ, TNF-α or IL-2. Cytokine responses presented are background subtracted values (from the frequency of cytokine produced by unstimulated cells).

### Quantification and statistical analysis

Statistical analyses were performed in Prism (v9.4.1; GraphPad Software Inc, San Diego, CA, USA). Non-parametric tests were used for all comparisons. The Mann-Whitney and Wilcoxon signed rank tests were used for unmatched and paired samples, respectively. Chi-square tests were used for comparisons between the proportion of responders represented as pie charts. All correlations reported are non-parametric Spearman’s correlations. Analysis of cytokine co-expressing populations was performed using SPICE version 5.1. p values less than 0.05 were considered statistically significant. Details of analyses performed for each experiment are described in the figure legends.
