# Supplementary material for: Distinct T cell polyfunctional profile in SARS-CoV-2 seronegative children associated with endemic human coronavirus cross-reactivity
Source: iScience. 2023 Dec 14;27(1):108728. doi: 10.1016/j.isci.2023.108728 (PMC10792240; doi:10.1016/j.isci.2023.108728)
Supplement: Document S1. Figures S1 and S2 [file mmc1.pdf]

iScience, Volume *27*

Supplemental information

Distinct T cell polyfunctional profile in SARS-CoV-2 seronegative children associated with endemic human coronavirus cross-reactivity

Ntombi Benede, Marius B. Tincho, Avril Walters, Vennesa Subbiah, Amkele Ngomti, Richard Baguma, Claire Butters, Lina Hahnle, Mathilda Mennen, Sango Skelem, Marguerite Adriaanse, Heidi Facey-Thomas, Christiaan Scott, Jonathan Day, Timothy F. Spracklen, Strauss van Graan, Sashkia R. Balla, Thandeka Moyo-Gwete, Penny L. Moore, Rae MacGinty, Maresa Botha, Lesley Workman, Marina Johnson, David Goldblatt, Heather J. Zar, Ntobeko A.B. Ntusi, Liesl Zühlke, Kate Webb, Catherine Riou, Wendy A. Burgers, and Roanne S. Keeton

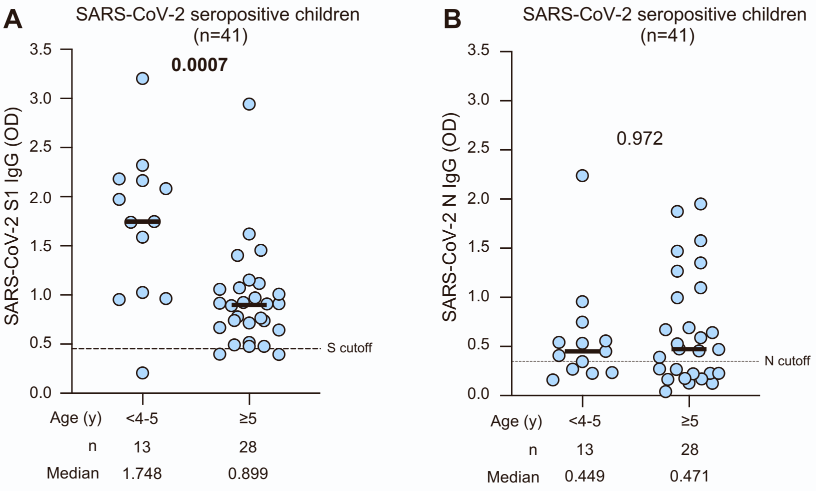

Supplementary Figure 1: SARS-CoV-2-specific antibody responses in children stratified by age, related to Figure 1.

(A) The magnitude of SARS-CoV-2 spike-specific IgG measured by ELISA in SARS-CoV-2 seropositive children younger than 5 years (n=13) and 5 years or older (n=28).

(B) The magnitude of SARS-CoV-2-nucleocapsid-specific IgG in in SARS-CoV-2 seropositive children younger than 5 years (n=13) and 5 years or older (n=28). Statistical comparisons in (A) and (B) were performed using the Mann-Whitney test between groups; p values <0.05 were considered statistically significant and are bolded.

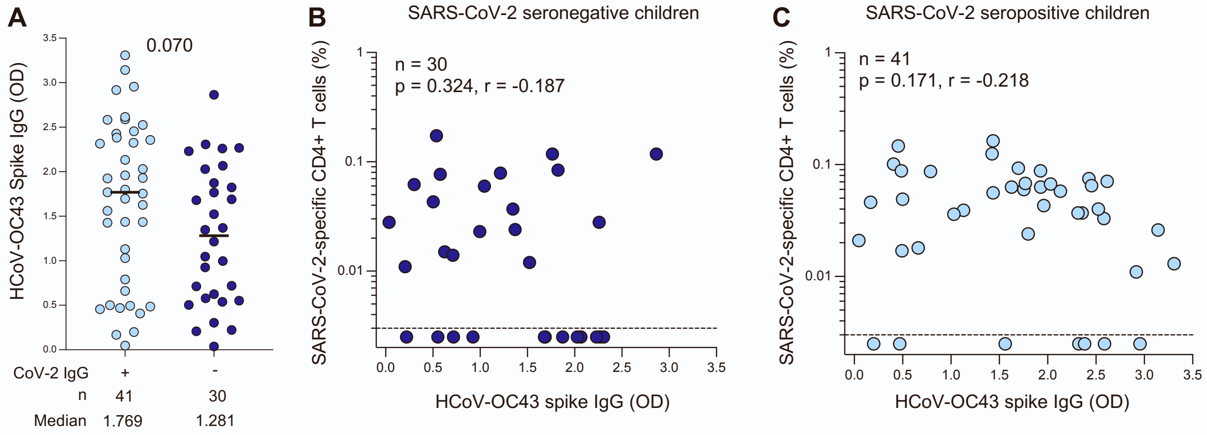

Supplementary Figure 2: SARS-CoV-2 cross-reactivity to endemic HCoV-OC43 in unvaccinated children, related to Figure 3.

(A) The magnitude of HCoV-OC43 spike IgG levels were measured by ELISA in SARS-CoV-2 seropositive (light blue; n=41) and seronegative (dark blue; n=30) children. The bars represent the median values. Statistical comparisons were performed using the Mann-Whitney test between seropositive and seronegative children; p values <0.05 were considered statistically significant.

(B) Correlation between the frequency of SARS-CoV-2-specific CD4+ T cells and HCoV-OC43-spike IgG levels in SARS-CoV-2 seronegative.

(C) Correlation between the frequency of SARS-CoV-2-specific CD4+ T cells and HCoV-OC43-spike IgG levels in SARS-CoV-2 seropositive children (n=41). Statistical comparisons in (B) and (C) were performed using a two-tailed non-parametric Spearman rank tests; p values <0.05 were considered statistically significant and are bolded and correlation coefficients values are shown.
